# Supplementary material for: Renewal of planktonic foraminifera diversity after the Cretaceous Paleogene mass extinction by benthic colonizers
Source: Nat Commun. 2022 Nov 21;13:7135. doi: 10.1038/s41467-022-34794-5 (PMC9681854; doi:10.1038/s41467-022-34794-5)
Supplement: Supplementary file 1 — Supplementary information [file 41467_2022_34794_MOESM1_ESM.pdf]

Supplementary information

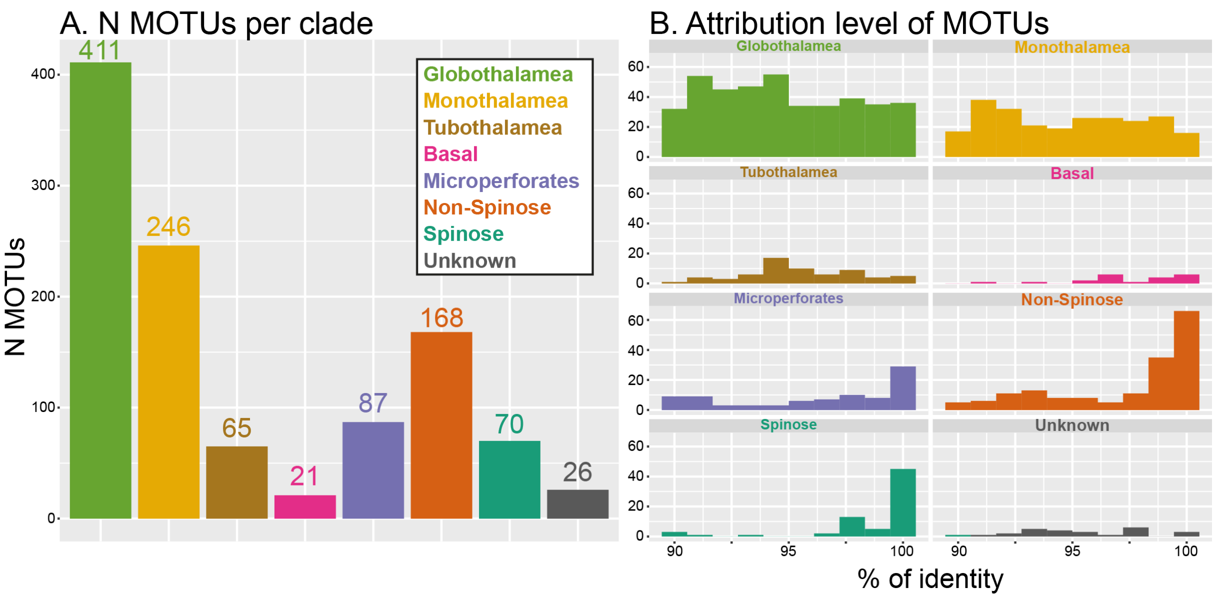

Supplementary Figure 1. Results of re-classification of the TARA Ocean dataset with updated taxonomic reference. **A.** Number of MOTUs attributed to each of the benthic and planktonic clades. **B.** Distribution of the percentage of identity of each clade against the reference database.

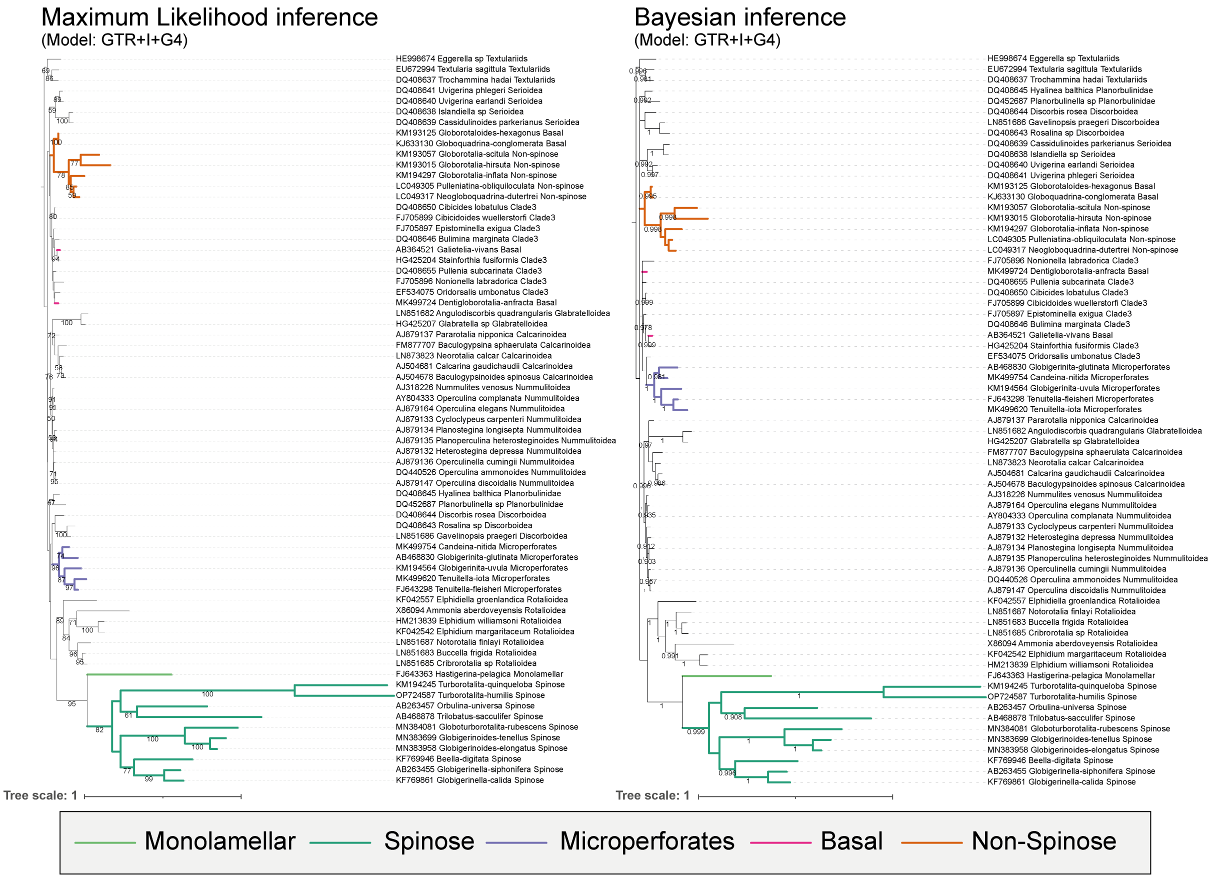

Supplementary Figure 2. Phylogenetic relationship of extant benthic Globothalamea with planktonic clades for the RAxML and Bayesian inferences. Each tree is rooted on the Textulariida.

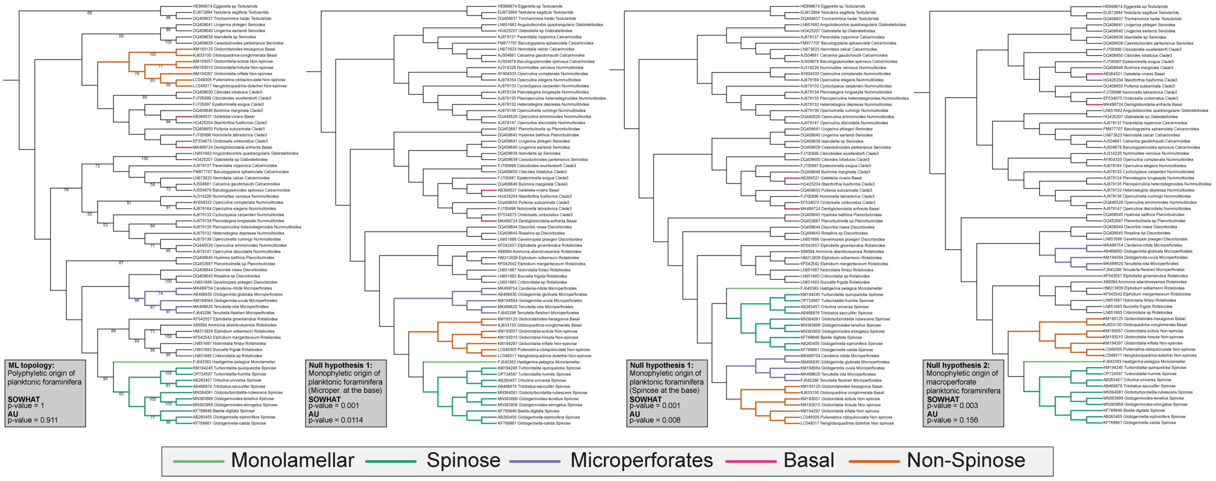

Supplementary Figure 3. Alternative phylogenetic hypotheses tested with the SOWHAT and AU test associated with their results.
