# Supplementary material for: Renewal of planktonic foraminifera diversity after the Cretaceous Paleogene mass extinction by benthic colonizers
Source: Nat Commun. 2022 Nov 21;13:7135. doi: 10.1038/s41467-022-34794-5 (PMC9681854; doi:10.1038/s41467-022-34794-5)
Supplement: Supplementary file 3 — Description of Additional Supplementary Files [file 41467_2022_34794_MOESM3_ESM.pdf]

Description of Additional Supplementary Files

File Name: Supplementary Data 1
Description: The updated version of the PR2_V9 database of Ref. 13 including an updated taxonomy of foraminifera.

File Name: Supplementary Data 2
Description: Occurrence table of planktonic foraminifera in individual TARA Ocean samples with their taxonomic affiliation according to the updated taxonomic database, and classification based on the dual Random Forest approach. The R code used for the Random Forest analysis is provided on Github: https://github.com/chassenr/ForamsOrigin.

File Name: Supplementary Data 3
Description: Alignment files and resulting topology for the backbone phylogeny together with the phylogenetic placement files presented on the Figure 3B.

File Name: Supplementary Data 4
Description: Alignment files and resulting topology placing the planktonic foraminifera within the Globothalamea clade for RAxML and MrBayes inferences. The phylogenetic tree of the Maximum Likelihood topology is shown as Fig. 4 associated with the posterior probability of Bayesian inference provided next to the branches, and the topologies with branch lengths are shown in Fig S2.

File Name: Supplementary Data 5
Description: Molecular clock trees estimating the divergence timing between foraminifera clades.
