# Supplementary figures and images for: Renewal of planktonic foraminifera diversity after the Cretaceous Paleogene mass extinction by benthic colonizers
Source: Nat Commun. 2022 Nov 21;13:7135. doi: 10.1038/s41467-022-34794-5 (PMC9681854; doi:10.1038/s41467-022-34794-5)

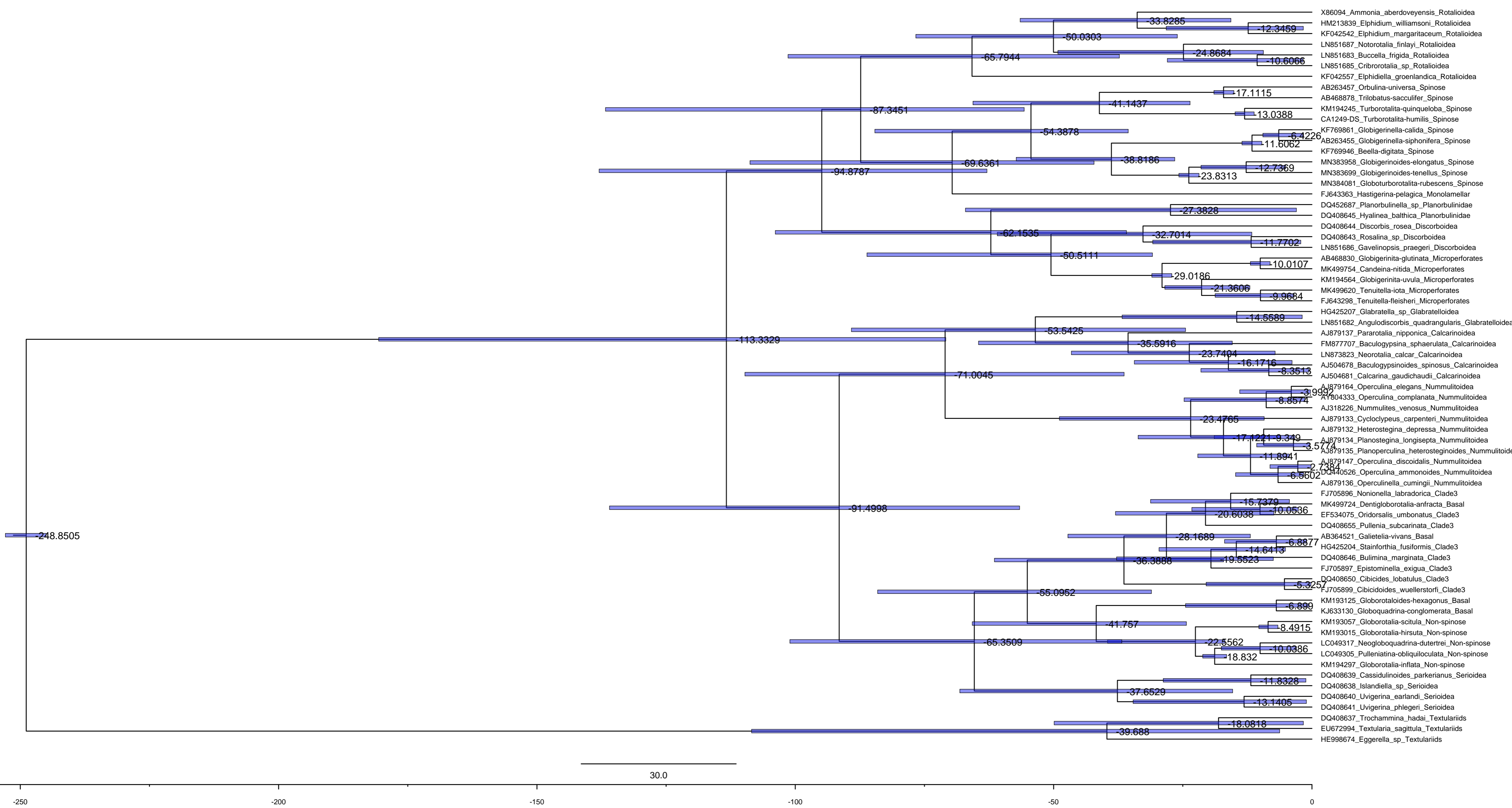

Supplement: Supplementary file 8 — Supplementary Data 5 [file 41467_2022_34794_MOESM8_ESM.zip › Supplementary Data 5/Molecular_clock_tree_Bayesian_Topology.pdf]

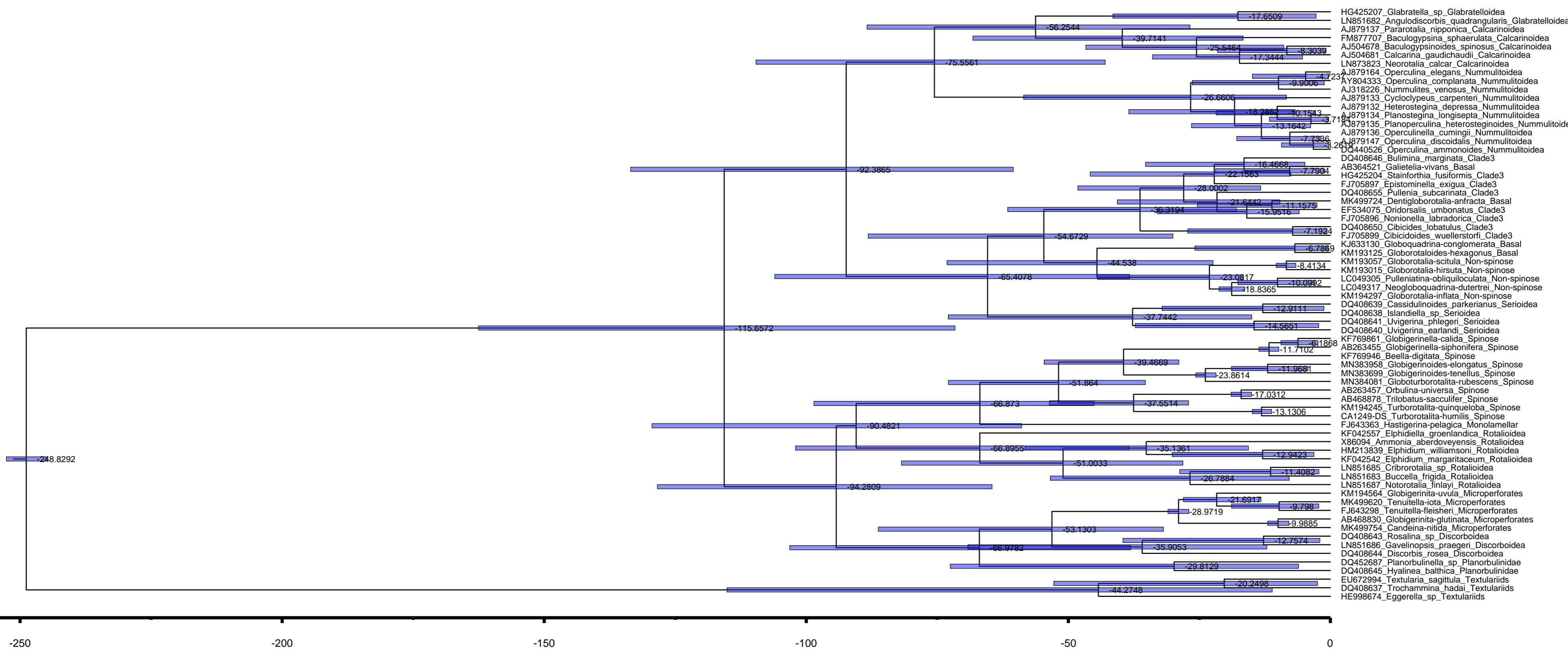

Supplement: Supplementary file 8 — Supplementary Data 5 [file 41467_2022_34794_MOESM8_ESM.zip › Supplementary Data 5/Molecular_clock_tree_ML_Topology.pdf]
