# Supplementary material for: Renewal of planktonic foraminifera diversity after the Cretaceous Paleogene mass extinction by benthic colonizers
Source: Nat Commun. 2022 Nov 21;13:7135. doi: 10.1038/s41467-022-34794-5 (PMC9681854; doi:10.1038/s41467-022-34794-5)
Supplement: Supplementary file 9 — Reporting Summary [file 41467_2022_34794_MOESM9_ESM.pdf]

nature portfolio

Corresponding author(s): Raphael Morard
Last updated by author(s): Oct 12, 2022

Reporting Summary

Nature Portfolio wishes to improve the reproducibility of the work that we publish. This form provides structure for consistency and transparency in reporting. For further information on Nature Portfolio policies, see our Editorial Policies and the Editorial Policy Checklist.

Statistics

For all statistical analyses, confirm that the following items are present in the figure legend, table legend, main text, or Methods section.

n/a	Confirmed	
☐	☒	The exact sample size (*n*) for each experimental group/condition, given as a discrete number and unit of measurement
☐	☒	A statement on whether measurements were taken from distinct samples or whether the same sample was measured repeatedly
☐	☒	The statistical test(s) used AND whether they are one- or two-sided<br>*Only common tests should be described solely by name; describe more complex techniques in the Methods section.*
☐	☒	A description of all covariates tested
☒	☐	A description of any assumptions or corrections, such as tests of normality and adjustment for multiple comparisons
☐	☒	A full description of the statistical parameters including central tendency (e.g. means) or other basic estimates (e.g. regression coefficient) AND variation (e.g. standard deviation) or associated estimates of uncertainty (e.g. confidence intervals)
☐	☒	For null hypothesis testing, the test statistic (e.g. *F*, *t*, *r*) with confidence intervals, effect sizes, degrees of freedom and *P* value noted<br>*Give P values as exact values whenever suitable.*
☐	☒	For Bayesian analysis, information on the choice of priors and Markov chain Monte Carlo settings
☒	☐	For hierarchical and complex designs, identification of the appropriate level for tests and full reporting of outcomes
☒	☐	Estimates of effect sizes (e.g. Cohen's *d*, Pearson's *r*), indicating how they were calculated

Our web collection on statistics for biologists contains articles on many of the points above.

Software and code

Policy information about availability of computer code

Data collection	no software used for collection
Data analysis	R version 4.0.2 using Rstudio version 1.2.1335; publicly available packages listed in method. The code used for the RandomForest analysis is provided on Github: https://github.com/chassenr/ForamsOrigin

For manuscripts utilizing custom algorithms or software that are central to the research but not yet described in published literature, software must be made available to editors and reviewers. We strongly encourage code deposition in a community repository (e.g. GitHub). See the Nature Portfolio guidelines for submitting code & software for further information.

Data

Policy information about availability of data

All manuscripts must include a data availability statement. This statement should provide the following information, where applicable:

- Accession codes, unique identifiers, or web links for publicly available datasets
- A description of any restrictions on data availability
- For clinical datasets or third party data, please ensure that the statement adheres to our policy

All data used and analyzed in the study are publicly available at ZENODO for the V9 TARA Ocean dataset (https://doi.org/10.5281/zenodo.3768509) and the database PR²_V9 (https://zenodo.org/record/3768951). The results of the reanalysis and/or modification of these data sources are available as part of the Supplementary Data files provided accompanying the manuscript.

Human research participants

Policy information about studies involving human research participants and Sex and Gender in Research.

Reporting on sex and gender	N/A
Population characteristics	N/A
Recruitment	N/A
Ethics oversight	N/A

Note that full information on the approval of the study protocol must also be provided in the manuscript.

Field-specific reporting

Please select the one below that is the best fit for your research. If you are not sure, read the appropriate sections before making your selection.

☐ Life sciences ☐ Behavioural & social sciences ☒ Ecological, evolutionary & environmental sciences

For a reference copy of the document with all sections, see nature.com/documents/nr-reporting-summary-flat.pdf

Ecological, evolutionary & environmental sciences study design

All studies must disclose on these points even when the disclosure is negative.

Study description	The study is a re-analysis of surface ocean metabarcoding data (TARA OCEANS) that shows the active dispersion of the Globothalamea benthic foraminifera in the plankton and serves to re-interpret the evolutionary history of planktonic foraminifera.
Research sample	The study is based on the TARA Oceans dataset that is publicly available at https://doi.org/10.5281/zenodo.3768509. The dataset was generated from sample collected in the surface Ocean during the TARA Ocean cruise to characterize pelagic living community below 2 mm body size.
Sampling strategy	The sampling strategy was to achieve a worldwide coverage of genetic diversity of plankton community and across several size fractions to collect data on all type of plankton. This was achieved through the deployment of plankton nets with various mesh size, water pumping and filtration to collect samples representative for the targeted communities. For a full description of sampling methodology: https://www.nature.com/articles/sdata201523
Data collection	The data collection was performed via the DNA extraction, PCR amplification and illumina sequencing of the V9 fragment of the SSU rDNA. For a full description of data collection and generation: https://www.sciencedirect.com/science/article/pii/S0092867419311249
Timing and spatial scale	The dataset is a global collection of oceanic surface water collected between September 2009 and June 2013.
Data exclusions	Only metabarcodes that could be reliably attributed to foraminifera using the modified version of the PR^2_V9 were retained in the study. See methods for further details.
Reproducibility	Reproducibility is ensured by the global coverage of the dataset.
Randomization	Randomization is ensured by the global coverage of the dataset.
Blinding	The generation of the dataset was done following a strict protocol that was defined independently of the authors of the study that could not influence the outcome of the results.

Did the study involve field work? ☐ Yes ☒ No

Reporting for specific materials, systems and methods

We require information from authors about some types of materials, experimental systems and methods used in many studies. Here, indicate whether each material, system or method listed is relevant to your study. If you are not sure if a list item applies to your research, read the appropriate section before selecting a response.

Materials & experimental systems

n/a	Involved in the study
☒	☐ Antibodies
☒	☐ Eukaryotic cell lines
☒	☐ Palaeontology and archaeology
☒	☐ Animals and other organisms
☒	☐ Clinical data
☒	☐ Dual use research of concern

Methods

n/a	Involved in the study
☒	☐ ChIP-seq
☒	☐ Flow cytometry
☒	☐ MRI-based neuroimaging
